# Scenario Studies on Effects of Soil Infiltration Rates, Land Slope, and Furrow Irrigation Characteristics on Furrow Irrigation-Induced Erosion

**DOI:** 10.1155/2014/942732

**Published:** 2014-11-10

**Authors:** Jibrin M. Dibal, A. A. Ramalan, O. J. Mudiare, H. E. Igbadun

**Affiliations:** ^1^Department of Agricultural and Environmental Resources Engineering, Faculty of Engineering, University of Maiduguri, PMB 1069, Maiduguri, Borno State, Nigeria; ^2^Department of Agricultural Engineering, Faculty of Engineering, Ahmadu Bello University, PMB 1044, Zaria, Nigeria

## Abstract

Furrow irrigation proceeds under several soil-water-furrow hydraulics interaction dynamics. The soil erosion consequences from such interactions in furrow irrigation in Samaru had remained uncertain. A furrow irrigation-induced erosion (FIIE) model was used to simulate the potential severity of soil erosion in irrigated furrows due to interactive effects of infiltration rates, land slope, and some furrow irrigation characteristics under different scenarios. The furrow irrigation characteristics considered were furrow lengths, widths, and stream sizes. The model itself was developed using the dimensional analysis approach. The scenarios studied were the interactive effects of furrow lengths, furrow widths, and slopes steepness; infiltration rates and furrow lengths; and stream sizes, furrow lengths, and slopes steepness on potential furrow irrigation-induced erosion, respectively. The severity of FIIE was found to relate somewhat linearly with slope and stream size, and inversely with furrow lengths and furrow width. The worst soil erosion (378.05 t/ha/yr) was found as a result of the interactive effects of 0.65 m furrow width, 50 m furrow length, and 0.25% slope steepness; and the least soil erosion (0.013 t/ha/yr) was induced by the combined effects of 0.5 l/s, 200 m furrow length, and 0.05% slope steepness. Evidently considering longer furrows in furrow irrigation designs would be a better alternative of averting excessive FIIE.

## 1. Introduction

The use of mathematical models and computer simulations in engineering, hydrology, and various fields in the process of decision making is progressively gaining more acceptances [1]. Models are useful tools for representing the detailed and complex “real world” with a more simple and understandable structure and can be used in demonstrating the relationships and interactions amongst various factors [2]. Furthermore, models allow the decision-maker to combine information from various sources and, in some cases, to extrapolate findings beyond the trial period [1]. They can also be employed in the identification of promising technologies and reduce the time and expense of field experiments by focusing resources on these most promising technologies [3].

Furrow irrigation is one of the most conventionally practiced irrigation methods in the area. About 60% of irrigated lands in Nigeria are furrow-irrigated [4]. Furrow irrigation is especially recommended for growing row crops on medium- to heavy-textured soils due to its simplicity and low capital costs [5]. Compared to traditional strip plot and other surface irrigation methods, furrow irrigation was found to have reduced water requirements by up to 28–43 percent [4]. However, one of the drawbacks of furrow irrigation is the soil erosion that it occasions. By this process, nutrient-rich top soil is removed, transported, and deposited somewhere, thereby constituting a nuisance. Soil erosion impacts negatively on both the environment and crop productivity and depresses the economic and social status of farmers [6]. Sojka et al. [7] reported 75% of Idaho furrow-irrigated fields lost their entire “A” horizon in the upper reaches together with a 2- to 4-fold increase in “topsoil” at the lower ends, reducing productivity by 25% over preerosion values and reducing yields by 20–50% in areas where top soil has been lost. Furrow irrigation-induced erosion (FIIE) has also become one of the major factors limiting factors to sustainable irrigated crop production in Samaru.

Erosion problems are complex owing to a combination of socioeconomic factors and common agricultural practices. A wide range of locally and internationally funded research projects from the government, nongovernmental organizations (NGOs), and the academia have addressed rainfall erosion problems in Samaru, for example, the works of Adewumi [8], Adewumi et al. [9], and Odunze [10]. But the predicament of FIIE has not received adequate attention. Unfortunately, the technologies and methods developed to understand, predict, and/or mitigate soil erosion that focused on rainfall-induced erosion cannot be effective on irrigation-induced erosion [11]. There is therefore a growing need for models to be applied in predicting, control, and understanding of FIIE. Leib et al. [12] pointed out that the minimum information needed to evaluate soil erosion in furrow includes the amount of soil lost during the irrigation along with some field and irrigation characteristics such as field slope, field length, soil texture, furrow inflow rate, and irrigation duration. Other useful information is furrow spacing, hydraulic radius, and soil-water continuum.

The* discharge measurements* which are the basis of FIIE assessment are commonly used to calculate the average soil loss per unit field area (Mg/ha) (Bjorneberg and Strelkoff, 2001). However, from a practical viewpoint, erosion rates on a field vary widely with many soil and furrow hydraulic factors. For example, erosion and sediment transport capacity increase with the shear or velocity of the flow, which in turn increase with the flow rate and furrow slope. Further, 50 to 80% of a typical furrow inflow infiltrates before it reaches the furrow end, resulting in a corresponding flow rate decrease along the furrows [13]. Understanding the interactions of soil and hydraulic factors affecting FIIE is indispensable for satisfactory decision making and management. It is obvious therefore that the future context of FIIE that an individual, an organization, or a development policy will have to respond to is both complex and uncertain. The ability to visualize and simulate support on problems related to land-soil-furrow hydraulics interactions through negotiating alternative practical possible combination of possible events or scenarios and proffer solution to them simultaneously is a vital tool in research toward averting FIIE.

Nicetic et al. [14] showed that scenarios studies can be used to generate knowledge about the present and the future and to identify the limits of that knowledge. It is also used as a public communication tool to draw the attention of the public to specific issues and for examining the potential effectiveness of a decision in solving a particular problem or understanding the potential dangers associated with interaction of working variables. Ajayi and Horta [15] used scenario analysis to investigate the effect of spatially varied soil hydraulic properties on runoff. This study aims at exploring the potential interactive effects of soil infiltration rates, slope steepness, and furrow irrigation characteristics on furrow irrigation-induced erosion in a semiarid region using a FIIE model. A brief of the model development is also presented.

## 2. Materials and Methods

### 2.1. Study Area

Field experiments were conducted during the 2009/2010 and 2010/2011 irrigation seasons at the Irrigation Research Field of the Institute for Agricultural Research (IAR) farm, Samaru, Zaria, along the Zaria-Sokoto road (11°1′N, 7°38′E, on the altitude of 686 m above mean sea level). Samaru is situated within the Northern Guinea savanna zone of Nigeria.

### 2.2. Experimental Treatments

The experimental factors studied were stream size *Q*, furrow length *L*, and furrow widths *W* at 3, 2, and 2 levels, respectively. The stream sizes were 2.5, 1.5, and 0.5 l/s; furrow lengths were 90 and 45 m; and furrow widths were 0.75 and 0.9 m. The combination of the stream sizes, furrow length, and furrow widths resulted in twelve (12) different treatments that were imposed on the field as follows:

(1) $$\begin{array}{c} T_{1}=Q_{1}L_{1}W_{1},\;\;T_{2}=Q_{1}L_{1}W_{2},\\ T_{3}=Q_{1}L_{2}W_{1},\;\;T_{4}=Q_{1}L_{2}W_{2},\\ T_{5}=Q_{2}L_{1}W_{1},\;\;T_{6}=Q_{2}L_{1}W_{2},\\ T_{7}=Q_{2}L_{2}W_{1},\;\;T_{8}=Q_{2}L_{2}W_{2},\\ T_{9}=Q_{3}L_{1}W_{1},\;\;T_{10}=Q_{3}L_{1}W_{2},\\ T_{11}=Q_{3}L_{2}W_{1},\;\;T_{12}=Q_{3}L_{2}W_{2}, \end{array}$$

where *Q*_1_, *Q*_2_, and *Q*_3_ are stream sizes 2.5, 1.5, and 0.5 l/s, respectively; *L*_1_ and *L*_2_ are furrow lengths 90 and 45 m, respectively; *W*_1_ and *W*_2_ are furrow widths 0.75 and 0.9 m. The layout of the experiment was a randomized complete block laid in a split plot design with four replications. This layout was observed in both 2009/2010 (Trial 1) and 2010/2011 (Trial 2) seasons. Main plots consisted of stream sizes while furrow lengths and furrow widths were focused in the subplots. In both of the study seasons, each replication comprised three plots, and each experimental plot had three and two ridges in Trials 1 and 2, respectively.

### 2.3. Field Measurements

#### 2.3.1. Preirrigation Data

The basic infiltration rate of the soil was determined using the inflow-outflow method. Slope of the field was determined in each of the two trials using the dumpy level surveying instrument. The hydraulic shear stress τ was obtained following the guidance of Schwab et al. [16]. The soil erodibility, *K*, was determined by adopting the universal soil loss equation (USLE) “*K*” equation as it is in Wall et al. [17]. The time of flow of water was measured directly from the field using a stopwatch.

#### 2.3.2. Irrigation/Erosion Related Data

Prior to commencement of irrigations, water-sediment collection stations were established 5 m before the end of each furrow. Flow of water in the furrows was measured using a cutthroat flume installed 5 m from entry at the upstream and at the tail end of the furrows for the measurement of water inflow and outflow of the furrows. Water flowing out of the furrows was measured as runoff. One liter of water-sediment samples was collected during irrigation events at each of the established measurement points and was taken to laboratory and filtered into preweighed metal containers. The resultant residues were oven-dried at 105°C over 24-hour period and reweighed. Sediment concentrations (g/L) were calculated from the dried residues and the runoff volumes for each of the treatments and were used to calculate soil erosion per furrow. Runoff volume was calculated as the product of the runoff discharge (l/s) (from the downstream flumes) and duration of runoff discharge. Soil erosion at the end of the furrows was calculated as the product of the sediment concentrations and runoff volumes divided by the wetted area. The wetted area was calculated as the product of the top width of flow of water and the length of the furrow [6, 18–20].

### 2.4. Model Development

The furrow irrigation-induced erosion (FIIE) model was developed using dimensional analysis approach following the concept of Buckingham *π*-theorem considering the variables in Table 1.

However, the principles of the Buckingham *π*-theorem require that the variables to be used in the dimensional analysis be theoretically independent. Some of the variables in (1) were therefore eliminated, and the resultant is the* decision variables* used in the model development as presented in Table 2.

A two-year field experiment was embarked upon to collect data for the completion of the model development. Five *π*-terms, namely, *π*_1_ to *π*_5_, were developed using the dimensional analysis and later combined to three. Three graphs were plotted, namely,  *π*_1_ against *π*_2_ keeping *π*_3_ constant, *π*_1_ against *π*_3_ keeping *π*_2_ constant, and *π*_1_ against *π*_2_ keeping *π*_3_ constant with supplementary data. Component equations were derived from the regression equations of the three graphs and they were further used to develop the model as presented in

(2) E=0.1110τI+0.4117TQ1/2τXWIK1/6 −0.0039τQK1/3XWI.

**Figure 1:**
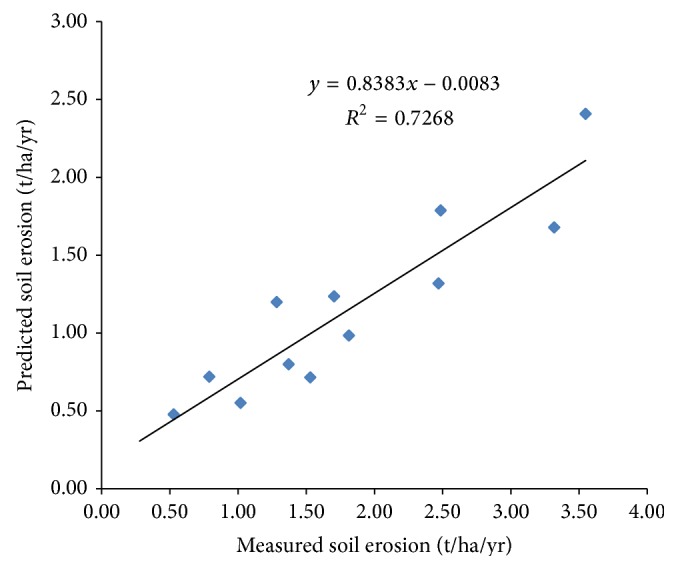
Comparison of predicted with measured soil erosion.

The validation of the model showed that the model's prediction has a 72.68% degree of accuracy (Figure 1). The efficiency criteria (ECs) [21, 22] used to study the error margin between the simulated and observed FIIE values showed the model has coefficient of variation (CV) of 0.412, Nash-Sutcliffe efficiency (NSE) of 0.7, and index of agreement (*D*) of 0.941. The model was found to be most sensitive to stream size and least sensitive to time of flow of water in the furrows.

### 2.5. Scenario Studies

Soil erosion in irrigated furrow is practically a function of many variables, among which only stream sizes, furrow length, and width were studied, and it is often not possible to monitor the influence of every land-use practice under all weather and soil conditions. Scientific planning for soil conservation and water management requires knowledge of the relations among those parameters that cause the soil erosion [23]. These parameters can be found by a careful analysis of the process or by controlled experimentation. Soil erosion predictions were therefore performed using the model and were used to rank alternative practices (scenarios) with regard to their likely cause and/or severity of soil erosion. In each of the scenarios, the selected values were inserted into the model and the resultant FIIE was computed using the model.

*Scenario 1: Effects of Furrow Lengths*,* Furrow Widths*,* and Slopes Steepness on Potential Furrow Irrigation-Induced Erosion*. According to Savva and Frenken [24], the width of the furrows varies from 45 to 65 cm, and the furrow spacing varies between 0.75 and 1.0 m, depending on soil type, crops, and stream size to be applied to the furrow. While coarse soils require closely spaced furrows in order to achieve lateral water flow in the root zone, clayey soils are comparatively spaced wider. These dynamics lead to variations in top width of flow and depth flow of water in the furrows during irrigation events. Conversely, typical furrow lengths vary from about 60 m on coarse textured soils to 500 m on fine textured soils, depending on the land slope, stream size, and irrigation depth. In areas where there is a considerable risk of erosion, the maximum slope should be limited to 0.3% [25]. Low irrigation efficiencies are usually associated with poor land leveling, erroneous stream size selection, and changes in soil type along the irrigated area both vertically and horizontally. However, the potential soil erosion corresponding to any combination of slope, top width of flow, and furrow length is still vague. But a large number of irrigations are going on with these types of combinations, implying a large potential of soil erosion threat. In this scenario study, to have a close representation of variation of top widths of flow due to changes of furrow widths, four furrow widths (0.65, 0.75, 0.85, and 0.95 m) were selected. Four furrow lengths (50, 100, 150, and 200 m) also selected have a near representation of various furrow lengths used by farmers in Nigeria. Slope steepness of 0.3, 0.5, 1, 1.5, 2, and 2.5% was considered to cover the acceptable slope steepness *S* in furrow irrigation.

*Scenario 2: Effect of Combined Infiltration Rates and Furrow Lengths on Potential Furrow Irrigation-Induced Erosion*. A number of soil factors affect farm irrigation system selection. Among other factors, soil texture plays a vital role through its effect on the available soil moisture and, most importantly, the infiltration rate of the soil. Many shallow soils, often with a depth of no more than 30 cm, are commonly developed for irrigation in many regions. At times, such soils may be of light texture obliging very frequent irrigation. The infiltration rate affects the length of run, size of furrows, and the application rates. Within the same soil textural group, infiltration rate can vary considerably, for instance, 20 to 30 and 5 to 10 mm/hr for sandy loam and clay loam soils, respectively [24]. Irrigation systems should be designed and managed in a way that the water application rate does not exceed the infiltrability of the soil. This scenario looks at potential erosion corresponding to furrow irrigation in various lengths of runs under varied infiltration rates.

*Scenario 3: Effects of Combination of Stream Sizes*,* Furrow Lengths*,* and Field Slopes on Potential Furrow Irrigation-Induced Erosion*. In irrigation practice, the use of very small stream size, *Q*, prolongs infiltration opportunity time (I.O.T.) and causes deep percolation at the upstream end of the furrow if the bottom end is also to receive enough water. On the other hand, the use of very large stream size speeds up the waterfront and reduces I.O.T.; this leads to high runoff losses and erosion hazards in the furrows. Apparently, the selection of appropriate combinations of stream sizes *Q* and furrow length *L* is necessary. The I.O.T. can be regulated in irrigated furrow to avert over- or underirrigation and its corresponding negative implications. Practically, this has not been of any concern to farmers despite its necessity in achieving good irrigation efficiency and averting erosion hazards. Water application can be regulated by increasing or decreasing the I.O.T. One can, within limits, regulate the depth of water applied. One method of achieving this is by decreasing the I.O.T. by steepening the slope *S* and shortening the furrow length *L* or by increasing the stream flow. In this method, one factor is varied while two other factors remain unchanged. But what is the magnitude of erosion problem associated with the combination of any factors mentioned? This scenario addresses it. In this analysis, three stream sizes (0.5, 1.5, and 2.5 l/s), four furrow lengths (50, 100, 150, and 200 m), and four slope steepness values (0.05, 0.3, 0.25, and 0.2%) were selected as representatives of what is commonly found in farmers' farm. For every value of *Q*, a corresponding value of *d* and *t* was measured from the field during the experiment and the values of *τ* were computed therefrom. The model was then used to generate the potential soil erosion values for every combination of *Q*, *L*, and *S*.  Table 3 gives some of the hydraulic properties measured.

## 3. Results and Discussion

### 3.1. Combined Effects of Furrow Lengths, Furrow Widths, and Slopes Steepness on Potential Furrow Irrigation-Induced Erosion

Table 3 contains the potential furrow irrigation-induced soil erosion resulting from different choices of furrow widths, lengths, and slopes. The table shows that, at any particular furrow width and length, there could be a very severe danger of soil erosion if there is any change in slope along the length of run. The highest soil erosion (378.05 t/ha/yr) was found as a result of the combined effects of irrigating in furrows that were 50 m long and 0.65 m wide on 0.25% steep fields. This prediction stresses the need of farm leveling to ensure uniform slope prior to commencement of irrigation. Practically, many farmers do not consider farm leveling as an important issue in irrigation, possibly due to illiteracy or fear of cost. The trend, however, cautioned against the practice of irrigation in short furrows irrespective of the slope in use.

### 3.2. Effect of Combined Infiltration Rates and Furrow Lengths on Potential Furrow Irrigation-Induced Erosion

The predicted FIIE as consequent combinations of furrow lengths and various infiltration rates is presented in Table 4. The table shows a diminishing trend of soil erosion with increase in furrow lengths at any particular infiltration rate. For example, at 20 mm/hr, soil erosion would dwindle by 59% between 50 and 200 m furrow lengths. This supports the possibility of irrigation in long furrows with little fear of severe soil erosion and counsels against irrigation in short furrows.

Similarly, the prediction shows another decreasing trend of soil erosion at a particular furrow length with increase in infiltration rate. For example, soil erosion dropped by about 33% in any particular furrow length between infiltration rates of 20 to 30 mm/hr. Often many irrigated fields, especially large farms, could have more than one type of soils or some slight spatial variation in the composition of the soil, hence varying in infiltration rates. The table supplies the irrigator with estimate possible of soil erosion that could be incurred and could be useful for planning irrigation.

### 3.3. Effects of Combination of Stream Sizes, Furrow Lengths, and Field Slopes on Potential Furrow Irrigation-Induced Erosion

Table 5 shows predicted furrow irrigation-induced erosion corresponding to practical combination of stream sizes, furrow lengths, and field slopes. The information that soil erosion increases with slope as it is in the table is not new; the table, however, provides the potential magnitude of soil erosion for any choice.

The prediction also called for prudence to be exercised when selecting large stream sizes to irrigate short furrows on steep slopes. When it is necessary to irrigate steep furrows, smaller stream size in a longer length of run is a better and safer option as pointed out in the table. The information in Tables 3 through 5 would serve as guide for the determination of optimum combinations of furrow lengths, widths, and stream sizes under varied slope and infiltration rates.

## 4. Conclusion

Models are employed in the systematic analysis of complex causal relationships to simulate the reactions and interactions of different variables to be able to identify inexplicit causal relationships of variables and their consequences. The FIIE model was herein used to explore the soil erosion consequences of the combinations of field slope, furrow lengths, top width of flow, infiltration rates, and stream sizes at various scales during furrow irrigation. The result stressed the danger of irrigating on steep slopped lands, cautioned against irrigation in short furrows, and called for prudent selection of flow stream sizes to circumvent severe soil erosion during furrow irrigation. Conducting such visualizations on a routine basis will help in providing a roadmap towards realization of sustainable irrigated agriculture.

## Figures and Tables

**Table 1:** Variables considered in the model development.

Variables	Symbols	Units	Dimensional symbols (M, L, T)
Soil erosion	*E*	Kg/m^2^/yr	ML^−2^T^−1^
Stream size	*Q*	m^3^/s	L^−3^T^−1^
Slope	*S*	%	[]
Furrow lengths	*X*	m	L
Top widths of flow	*W*	m	L
Hydraulic radius	*R*	m	L
Infiltration rate	*I*	mm/hr	LT^−1^
Time of flow	*T*	sec	T
Flow velocity	*V*	m/s	LT^−1^
Manning's roughness coefficient	*n*	sec/m^1/3^	TL^−1/3^
Acceleration due to gravity	*g*	m/s^2^	LT^−2^
Density of water	*ρ*_*w*_,	kg/m^3^	ML^−3^
Soil particle density	*ρ*_*s*_,	kg/m^3^	ML^−3^

**Table 2:** Decision variables and their corresponding dimensions.

Variables	Symbols	Units	Dimensional symbols (M, L, T)
Soil erosion	*E*	kg/m^2^/yr	ML^−2^T^−1^
Stream size	*Q*	m^3^/s	L^3^T^−1^
Furrow length	*X*	m	L
Top width of flow	*W*	m	L
Soil infiltration rate	*I*	mm/hr	LT^−1^
Time of flow of water	*T*	s	T
Soil erodibility factor	*K*	Kg·hr/Nm^2^	L^−3^T^3^
Hydraulic shear stress	*τ*	N/m^2^	ML^−1^T^−2^

**Table 3:** Potential furrow irrigation-induced erosion (t/ha/yr) at combined furrow lengths, furrow widths, and slopes steepness.

Furrow widths (m)	Furrow lengths (m)	Slopes (%)				
		0.05	0.1	0.15	0.2	0.25
		Furrow irrigation-induced erosion (t/ha/yr)				
0.65	50	75.6111	151.3	226.83	302.44	378.05
	100	38.8	77.6	116.40	155.20	194.00
	150	26.52	50.06	79.59	106.12	132.65
	200	20.4	40.8	61.19	81.58	101.98
0.75	50	65.79	131.59	197.38	263.18	328.97
	100	33.89	67.78	101.68	135.57	169.46
	150	23.26	46.52	69.78	93.04	116.30
	200	17.94	35.88	53.82	71.77	89.71
0.85	50	58.28	116.56	174.85	233.13	291.42
	100	30.13	60.27	90.41	120.54	150.68
	150	20.75	41.50	62.26	83.01	103.77
	200	16.06	32.12	48.18	64.25	80.31
0.95	50	52.34	104.69	157.04	209.39	261.74
	100	27.16	54.33	81.50	108.67	135.84
	150	18.77	37.55	56.32	75.10	93.87
	200	14.57	29.15	43.73	58.31	72.89

**Table 4:** Potential furrow irrigation-induced erosion (t/ha/yr) at combined infiltration rates and furrow lengths.

Furrow lengths (m)	Basic infiltration rates (mm/hr)				
	20	22	24	28	30
50	1.867	1.697	1.556	1.333	1.244
100	1.134	1.031	0.945	0.810	0.756
150	0.890	0.809	0.742	0.636	0.594
200	0.768	0.698	0.640	0.549	0.512

**Table 5:** Predicted furrow irrigation-induced erosion (t/ha/yr) corresponding to combination of stream sizes, furrow lengths, and field slopes.

Stream size (l/s)	Furrow lengths (m)	Slope (%)			
		0.05	0.10	0.15	0.20
0.5	50	0.024	0.047	0.071	0.095
	100	0.016	0.033	0.049	0.066
	150	0.014	0.027	0.041	0.054
	200	0.013	0.026	0.039	0.052

1.5	50	0.030	0.061	0.091	0.121
	100	0.019	0.039	0.058	0.078
	150	0.015	0.030	0.045	0.060
	200	0.014	0.028	0.043	0.057

2.5	50	0.043	0.086	0.128	0.171
	100	0.027	0.053	0.080	0.106
	150	0.020	0.040	0.060	0.080
	200	0.019	0.038	0.056	0.075
